# Hypoxia Promotes Glioma Stem Cell Proliferation by Enhancing the 14-3-3*β* Expression via the PI_3_K Pathway

**DOI:** 10.1155/2022/5799776

**Published:** 2022-05-14

**Authors:** Weidong Cao, Qiang Zhou, Hongwei Wang, Wei Rao, Gang Cheng, Peng Wang, Shengli Guo, Bin Ren, Jianning Zhang

**Affiliations:** ^1^Department of Neurosurgery, The First Medical Centre, Chinese PLA General Hospital, China; ^2^Department of Orthopedics, The Air Force Hospital of Eastern Theater, China

## Abstract

Glioma is a serious fatal type of cancer with the shorter median survival period and poor quality of living. The overall 5-year survival rate remains low due to high recurrence rates. Glioma stem cells (GSCs) play the important roles in the development of gliomas. Examination of the numerous biomarkers or cancer-associated genes involved in the development or prevention of glioma may therefore serve the discovery of novel strategies to treat patients with glioma. Hypoxia induced by using CoCl_2_ application and 14-3-3*β* protein knockdown by specific small interfering RNA transfection were performed in GSCs both in vitro and in vivo to observe their role in glioma progression and metastasis occurrence by using western blot analysis and MTT assay. The results demonstrated that CoCl_2_ application enhanced the 14-3-3*β* protein expression and mRNA levels via the PI_3_K pathway in GSCs. Furthermore, hypoxia promoted GSC cell proliferation and activated the expression of proliferating cell nuclear antigen, which was inhibited following 14-3-3*β* knockdown. In addition, tumor growth in mice was enhanced by CoCl_2_ application but reversed following 14-3-3*β* knockdown, which also enhanced GSC cell apoptosis. In conclusion, the present study demonstrated that hypoxia promoted glioma growth both in vitro and in vivo by increasing the 14-3-3*β* expression via the PI_3_K signaling pathway. 14-3-3*β* and HIF-1*α* may therefore be considered as the potential therapeutic target to treat patients with glioma.

## 1. Introduction

Glioma is a serious fatal type of tumor with a shorter median survival period and poor quality of living, and grade IV glioblastoma (GB) is the most malignant type of glioma [1–3]. Abnormal gene expression or mutation including tumor-suppressor inactivation or oncogene activation could be the certain genetic reasons for the development of gliomas. In addition, more recent evidence has been proved that glioma stem cells (GSCs), exhibiting stem cell-like self-renewal capabilities, involved in the process of initiation of tumors and played the important roles in the development of gliomas [4, 5]. Although progress has been obtained in the last 20 years in the development of numerous clinical treatments for glioma patients, the overall 5-year survival rate remains low due to high recurrence rates [6, 7]. Thus, the novel therapeutic strategies for glioma patients are urgent and to also understand the underlying mechanism of recurrence to tailor specific treatment. Previous studies reported that numerous biomarkers or cancer-associated genes, which could be potential targets, serve crucial roles in the development or prevention of glioma, and that these novel targets will be developed as antitumor agents [8–10].

It has been demonstrated that hypoxia inducible factor (HIF), including HIF-1*α* and HIF-1*β* subunits, is a heterodimeric transcription factor which may involve in the tumor angiogenesis and progression [11, 12]. HIF-1*α* regulated numerous physiological functions and even tumorigenesis in the hypoxic tumor microenvironment (TME) [13, 14] and modulated the tumor development and survival by involving in the tumor cell apoptosis, cell proliferation, differentiation, or migration, which serves a crucial role in the progression of breast, esophageal, lung, prostate, colon, and liver cancer [15–17]. Some studies reported that HIF-1*α* inhibition could induce the changes in radio- or chemosensitivity and angiogenesis [18, 19]. Our previous studies also evidenced that the malignant progression of glioma was related to the HIF-1*α* and vascular endothelial growth factor (VEGF) [20], and silencing of HIF-1*α* by siRNA application could enhance the radiation sensitivity of human glioma growth in vitro and in vivo [21]. Generally, certain molecular pathways, including phosphoinoisitide dependent kinase l, mTOR-RICTOR kinases and PI_3_K-AKT, could be affected or controlled by HIF-1*α*, which is itself an attractive drug target for clinical tumor treatment [22, 23]. In addition, HIF-1*α* was associated with the tumor angiogenesis and progression of glioma by regulating the apoptosis-related protein expression through the PTEN/Akt pathway [24, 25]. HIF-1*α* combined with JAK1/2-STAT3 (Janus kinase 1/2-signal transducer and activator of transcription 3) axis could enhance the self-renewal of glioma stem-like cells [26]. Clinical study also demonstrated that high hypoxia signature was associated with poor prognosis for glioma patients [27]. And amplification of EGFR activated the PI3K pathway in approximately 45% of GBM cases [28], and the p85/PI3K regulatory effect has been obtained in approximately 15% of GBM patients [29]. Furthermore, multitherapy that would include HIF-1*α* inhibition combined with chemotherapeutic regimens, radiotherapy, or other cancer gene-targeted reagents may be beneficial in clinical cancer therapy [30, 31].

Proteins 14-3-3 include seven distinct isoforms and constitute a family of acidic polypeptides found in mammals [32, 33]. There is strong evidence that the 14-3-3 overexpression promotes tumor growth and progression in glioma. 14-3-3*β* affects a wide range of biological processes, including signal transduction, DNA replication, DNA repair, cell cycle control, and vesicular transport, by binding to phosphoserine-containing sequence motifs in different pathways [34–36]. Previous studies demonstrated that 14-3-3*β*, which is considered as an oncogene and serves a crucial role in tumor angiogenesis and tumor formation, can modulate tumor cell proliferation, motility, cycle, and apoptosis via specific stimulator factors or downstream pathways, including mitogen-activated protein kinase- (MAPK-) dependent signal activation [37, 38].

Although several clinical studies reported that both 14-3-3*β* overexpression and HIF-1*α* are associated with the pathological characteristics and progression of various types of tumor [21, 32, 33], few study has reported the association between HIF-1*α* and 14-3-3*β* modulation in tumor progression and metastasis, notably in glioma. The present study is aimed therefore at clarifying the underlying mechanism of HIF-1*α* promoted glioma growth in vivo and in vitro by enhancing the 14-3-3*β* expression, which activates the downstream PI_3_K pathway. Effective inhibition on HIF-1*α* using novel inhibitors combined with 14-3-3*β* knockdown may be considered as a potential therapeutic strategy to treat patients with glioma.

## 2. Materials and Methods

### 2.1. Cell Culture

For GSC culture, clinical samples from patients were prepared as described in our previous reports [39, 40] with serum free Dulbecco's Modified Eagle's Medium (DMEM)/F12 (Invitrogen) including epidermal growth factor (EGF, 20 ng/mL), basic fibroblast growth factor (bFGF, 20 ng/mL) and B27 (1 : 50), and placed at 37°C in a humidified incubator containing 5% CO_2_.

### 2.2. Small Interfering (si) RNA Transfection

GSCs were cultured and transfected with 100 nM 14-3-3*β* siRNA and control vector (Qiagen, Inc.) using Lipofectamine™ 2000 Transfection Reagent (Invitrogen; Thermo Fisher Scientific, Inc.). The fresh medium was changed after 24 h following transfection, and the transfection efficiency was verified by western blotting after 48 h. The 14-3-3*β* targeting sequences were as follows: forward *5*′*-ATTGAGGAGGTCACAGAGAAC-3*′ and reverse *5*′*-TTCATATCCTGTTTTGGCCTG-3*′.

### 2.3. Western Blotting

The expression of 14-3-3*β* in GSCs was first evaluated by western blotting after chloride hexahydrate (CoCl_2_) application (10, 50, and 150 *μ*M) or 14-3-3*β* siRNA transfection for 48 h. GSCs were lysed using lysis buffer (100 mg/mL PMSF, 1% NP-40, 0.01 M Tris-HCl (pH 7.6), 1 mM EDTA (pH 8.0), and 1% (w/v) Triton X-100), and above chemicals were purchased from Sigma-Aldrich. Then, total proteins were obtained following cell lysate centrifugation at 12,000 x g for 30 min at 4°C. Protein concentration was measured by bicinchoninic acid assay (Thermo Fisher Scientific, Inc.). After that, proteins were separated by SDS-PAGE (4% stacking gel and 12% resolving gel) and transferred onto polyvinylidene fluoride membranes. Membranes were blocked by 5% BSA (Sigma-Aldrich) for 1 h at 37°C and incubated with the primary antibodies against 14-3-3*β* (1 : 1,000), HIF-1*α* (1 : 1,000), and *β*-actin (1 : 1,000; all Santa Cruz Biotechnology, Inc.) overnight at 4°C, respectively. Membranes were then incubated with a horseradish peroxidase–conjugated secondary antibody for 2 h at room temperature. Enhanced chemiluminescence (Thermo Fisher Scientific, Inc.) was used to detect the signal on the membrane. Data were analyzed via densitometry using ImageJ (Version 1.8.0; National Institutes of Health, Bethesda, MD, USA). In addition, the protein expression of proliferating cell nuclear antigen (PCNA), which is a typical biomarker of cell proliferation [41, 42], was also detected by western blotting according to above protocol (anti-PCNA, 1 : 1,000; Santa Cruz Biotechnology, Inc.).

### 2.4. Cell Proliferation Assay

GSCs were harvested by trypsinization and cultured in triplicate in 96-well plates (2 × 10^3^ cells per well). MTT assay (Roche Diagnostics) was used to evaluate cell proliferation following CoCl_2_ treatment (50 *μ*M for 24 h) or 14-3-3*β* siRNA transfection after incubation with 10 *μ*l MTT for 2 h. Absorbance was measured with a microplate spectrophotometer at 570 nm.

### 2.5. Real-Time PCR Analysis

Next, the mRNA levels were also detected by using real-time quantitative PCR (qPCR) analysis following the manufacturer's instructions. Total RNA was isolated by using TRIzol Reagent (Invitroge*n*). The ABI Prism 7500 real time PCR system (Applied Biosystems) was used to evaluate mRNA changes following the above treatment and siRNA transfection.

### 2.6. In Vivo Tumor Xenograft Model

Mice were aged six to eight weeks old and half for male or female in each group. GSCs (control or 14-3-3*β* siRNA transfected cells) were injected into the left dorsal flank of nude mice to set up the animal model with subcutaneous tumor xenografts. Mice were then treated with 5 mM CoCl_2_ by subcutaneous injections 4 weeks following GSC injection. Mice were anesthetized prior to injection and executed via an approved method of euthanasia after the treatment. The research protocol was approved by the Animal Ethics Committee. Tumor volumes were measured every 7 days according to the following equation: *V* = *L* × *W*^2^ x *π*/6, where *V* is the tumor volume, *L* is the tumor length, and *W* is the tumor width. Kaplan-Meier analysis was used to calculate the animal survival rates in order to evaluate the potential therapeutic effects of CoCl_2_ and/or 14-3-3*β* siRNA transfection [43–45].

### 2.7. Cell Apoptosis Analysis

After CoCl_2_ application or 14-3-3*β* siRNA transfection, GSC cell apoptosis rate was evaluated by annexin V-fluorescein isothiocyanate and propidium iodide staining (BD Biosciences) for 15 min at RT (25°C) in the dark and flow cytometry analysis (BD, FACSuite Research Assay Software). Bax/Bcl-2 assessment is a way of assessing cell apoptosis [46, 47]. Thus, Bax/Bcl-2 protein expression and mRNA levels were also detected by western blotting or qPCR following 14-3-3*β* siRNA transfection combined with CoCl_2_ treatment (anti-Bax and anti-Bcl-2, 1 : 1,000; Santa Cruz Biotechnology, Inc.). In addition, caspase-3 activity was measured to determine GSC cell apoptosis after above treatments by using a kit (Promega, Inc.) according to the manufacturer's instructions [48].

### 2.8. PI_3_K Effect

To test the effect of PI_3_K on 14-3-3*β* siRNA transfection, the specific PI_3_K pan isoform inhibitor *TG 100713* (Tocris Bioscience) was used at 10 *μ*M to treat GSCs for 48 h prior to CoCl_2_ application [49]. Subsequently, the 14-3-3*β* protein expression was also detected using western blotting.

### 2.9. Statistical Analysis

Statistical analysis was performed using Student's *t*-test and one-way ANOVA followed by a Tukey post hoc test. *P* < 0.05 was considered to indicate a statistically significant difference. Data were expressed as means ± standard deviation.

## 3. Results

### 3.1. Effect of Hypoxia on the 14-3-3*β* Expression in GSCs

**Figure 1:**
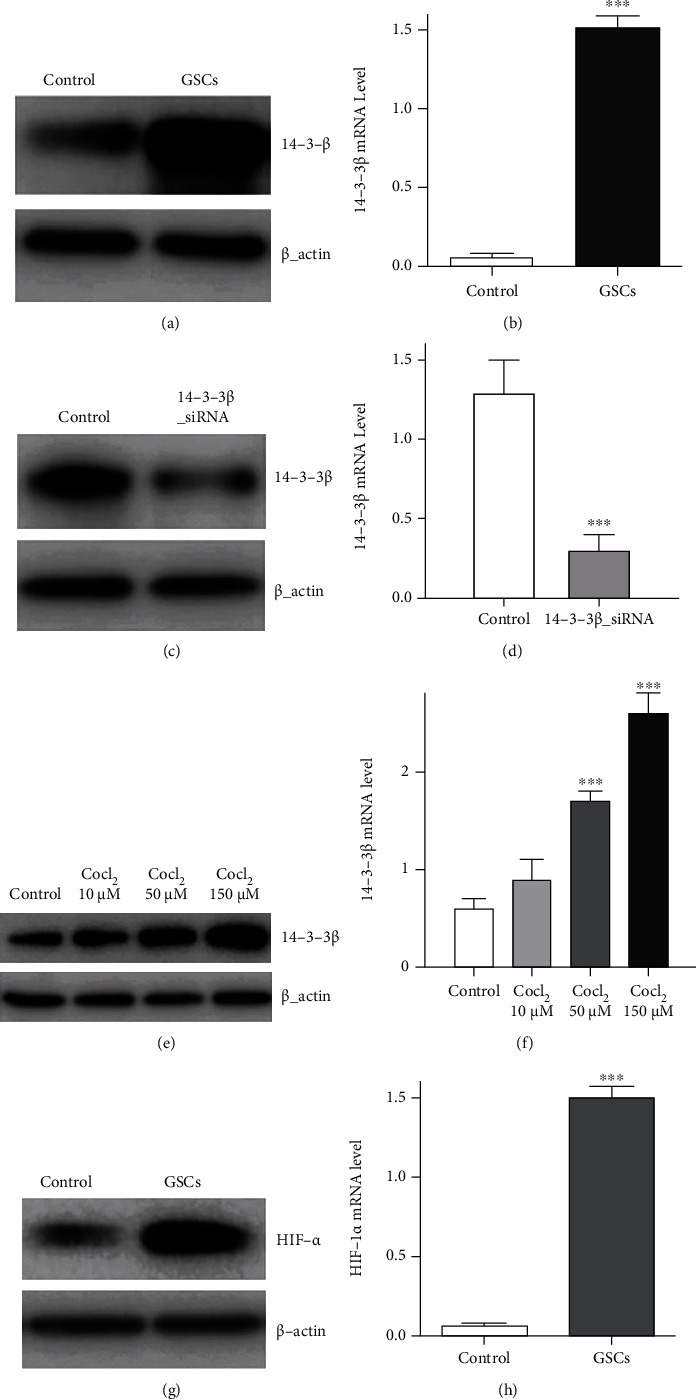
HIF-1*α* and 14-3-3*β* expression in GSCs. (a) 14-3-3*β* protein expression in GSC cells. (b) 14-3-3*β* mRNA level in GSC cells (vs. control, *P* < 0.001). (c) Effect of 14-3-3*β* siRNA transfection on the 14-3-3*β* expression in GSC cells. (d) Effect of 14-3-3*β* siRNA transfection on 14-3-3*β* mRNA level in GSCs cells (*14-3-3β siRNA* vs. control, *P* < 0.001). (e) Effect of hypoxia induced by using CoCl_2_ on the 14-3-3*β* protein expression in GSC cells. (f) Effect of hypoxia on 14-3-3*β* mRNA level in GSC cells (CoCl_2_ 50 *μ*M vs. control, *P* < 0.001; CoCl_2_ 150 *μ*M vs. control, *P* < 0.001). (g) HIF-1*α* expression in GSC cells. *β*-Actin was used as a loading control. (h) HIF-1*α* mRNA level in GSCs cells (vs. control, *P* < 0.001).

The expression of 14-3-3*β* and HIF-1*α* in GSCs was determined by western blotting and qPCR. The high expression of 14-3-3*β* and HIF-1*α* protein was observed in GSCs. In addition, 14-3-3*β* and HIF-1*α* mRNA levels were significantly increased in GSCs (Figure 1).

To observe the effect of 14-3-3*β* knockdown, specific 14-3-3*β* siRNA was transfected into GSCs to reduce the 14-3-3*β* expression firstly. The transfection efficiency of 14-3-3*β* siRNA was evaluated by qPCR and western blotting. The result confirmed that the 14-3-3*β* expression in GSCs was inhibited after 14-3-3*β* siRNA transfection for 48 h. In addition, 14-3-3*β* mRNA level was also significantly reduced after 14-3-3*β* siRNA application (Figure 1). These results provided the direct evidence that using 14-3-3*β* siRNA could reduce the 14-3-3*β* expression in GSCs significantly.

It has been demonstrated that hypoxia enhanced the HIF-1*α* expression [13, 16], and thus the effect of hypoxia induced by using CoCl_2_ on the 14-3-3*β* expression was subsequently investigated in GSCs too. To do so, GSCs were incubated with 10, 50, and 150 *μ*M CoCl_2_ for 8 h prior to mRNA level evaluation, respectively. Furthermore, cells were treated with different concentrations (10, 50, and 150 *μ*M) of CoCl_2_ for 24 h to observe the changes on the 14-3-3*β* expression. The results from western blotting and qPCR demonstrated that GSC treatment with CoCl_2_ could increase the 14-3-3*β* protein expression and mRNA level in a dose-dependent manner. The above results also confirmed that both 14-3-3*β* and HIF-1*α* were overexpressed in GSCs and that hypoxia enhanced the 14-3-3*β* expression (Figure 1).

### 3.2. Effects of 14-3-3*β* Knockdown on GSC Cell Proliferation

**Figure 2:**
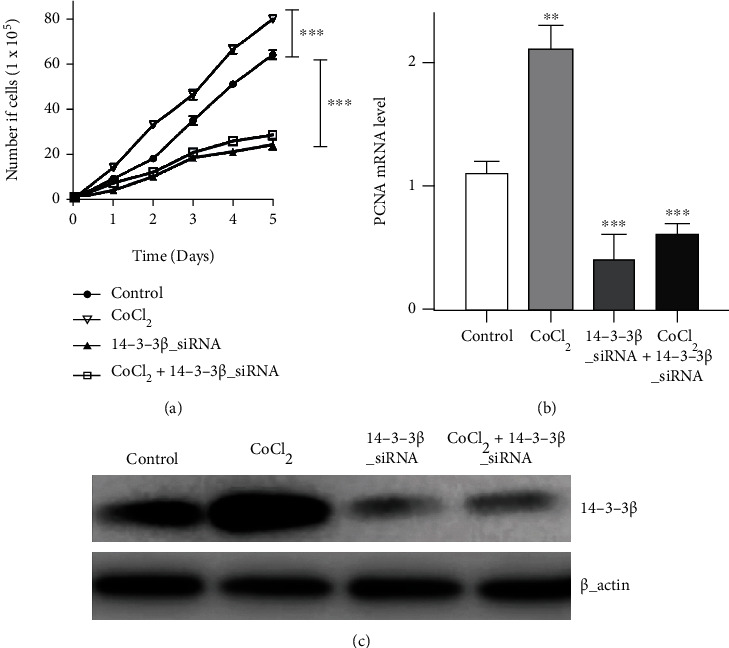
Effects on cell proliferation. (a) Effects of hypoxia and 14-3-3*β* siRNA transfection on GSC cell proliferation assessed by MTT assay (14-3-3*β* siRNA vs. control, *P* < 0.001; CoCl_2_ vs. control, *P* < 0.001). (b) Effects of CoCl_2_ application and 14-3-3*β* siRNA transfection on PCNA mRNA level in GSC cells (CoCl_2_ vs. control, *P* < 0.01; 14-3-3*β* siRNA vs. control, *P* < 0.001; CoCl_2_+14-3-3*β* siRNA vs. *CoCl_2_,P* < 0.001). (c) Effects of CoCl_2_ application and 14-3-3*β* siRNA transfection on the PCNA protein expression in GSC cells. *β*-Actin was used as a loading control.

Our previous study reported that inhibition on HIF-1*α* could be important for the biological behavior changes of glioma cells [21]. In the current experiments, GSC cell proliferation was therefore evaluated by MTT assay following CoCl_2_ application and 14-3-3*β* siRNA transfection. The results demonstrated that, in the group treated with 14-3-3*β* siRNA alone, cell proliferation was significantly reduced compared with that of the untreated group, and that cell count was reduced by 38.2%; however, a noticeable enhancement in cell numbers was obtained using CoCl_2_ alone. Notably, 14-3-3*β* knockdown by siRNA reversed the effect of CoCl_2_ on cell proliferation, and GSC cell count decreased by 31.3% following CoCl_2_ application combined with 14-3-3*β* siRNA transfection (Figure 2).

Furthermore, to observe the changes on cell proliferation after 14-3-3*β* knockdown, the expression of PCNA in GSCs was determined by western blotting and qPCR following CoCl_2_ and/or 14-3-3*β* siRNA application. Hypoxia induced by using CoCl_2_ enhanced PCNA protein expression and mRNA levels compared with the control group in GSCs (Figure 2). In addition, the PCNA protein expression in GSCs was reduced after 14-3-3*β* siRNA transfection, and PCNA mRNA levels were significantly inhibited by 14-3-3*β* siRNA application. And the reverse effect was also obtained in the group of CoCl_2_ application, and the PCNA protein and mRNA levels were decreased by combining CoCl_2_ treatment with 14-3-3*β* siRNA.

### 3.3. Effects of 14-3-3*β* Knockdown on Subcutaneous Tumor Growth

**Figure 3:**
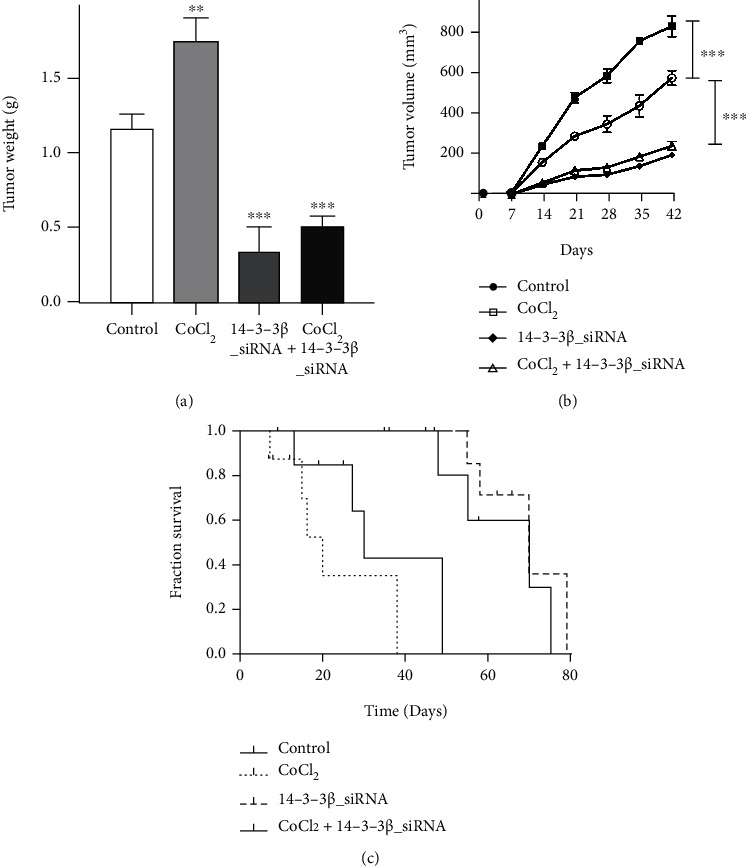
Effects of 14-3-3*β* knockdown on subcutaneous tumor growth. (a) Effect of CoCl_2_ application and 14-3-3*β* siRNA transfection on tumor weight in a tumor xenograft model (CoCl_2_ vs. control, *P* < 0.01; 14-3-3*β* siRNA vs. control, *P* < 0.001; CoCl_2_+14-3-3*β* siRNA vs. CoCl_2_, *P* < 0.001). (b) Effect of CoCl_2_ application and 14-3-3*β* siRNA transfection on tumor volume in a tumor xenograft model (14-3-3*β* siRNA vs. control, *P* < 0.001; CoCl_2_ vs. control, *P* < 0.001). (c) Effect of CoCl_2_ and 14-3-3*β* siRNA transfection on mice survival rate.

An in vivo tumor xenograft study was performed to evaluate the animal survival rate and tumor volume. The tumor weight in each group was also measured. The results demonstrated that CoCl_2_ increased tumor weight, whereas 14-3-3*β* knockdown significantly reduced it. In the control group, the tumor volume was 350 ± 41 mm^3^ following injection for 28 days and increased to 588 ± 33 mm^3^ when treated with CoCl_2_. Conversely, tumor volume was reduced to 100 ± 12 mm^3^ after 14-3-3*β* siRNA application. In addition, the enhancing effect of CoCl_2_ application on tumor volume was reversed, and tumor volume reached 135 ± 8 mm^3^ (Figure 3) in the combined group. These results indicated that 14-3-3*β* knockdown could inhibit tumor growth, and hypoxia induced by using CoCl_2_ increases the tumor growth which could be also reversed by combining 14-3-3*β* siRNA transfection.

The survival rate of transplanted mice was also determined following CoCl_2_ application and 14-3-3*β* knockdown. The results demonstrated that 14-3-3*β* siRNA application markedly prolonged mice survival rate; however, a lower survival rate was observed after CoCl_2_ treatment which inducing the hypoxic tumor microenvironment (TME) (Figure 3). 14-3-3*β* knockdown reduced tumor growth and prolong mice survival rate, and such 14-3-3*β* inhibition could reverse the effect of hypoxia on tumor growth in vivo.

### 3.4. Effects of 14-3-3*β* Knockdown on Cell Apoptosis

**Figure 4:**
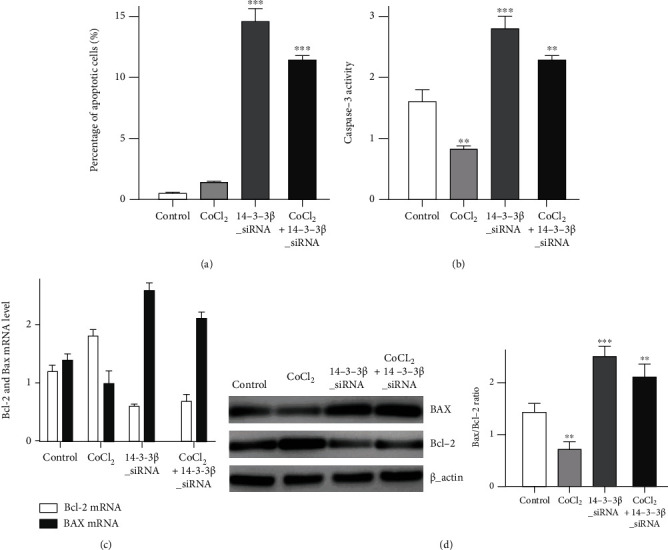
Effects on GSC cell apoptosis. (a) Effects of CoCl_2_ application and 14-3-3*β* siRNA transfection on the apoptotic rate of GSCs (14-3-3*β* siRNA vs. control, *P* < 0.001; CoCl_2_+14-3-3*β* siRNA vs. CoCl_2_, *P* < 0.001). (b) Effects of CoCl_2_ application and 14-3-3*β* siRNA transfection on caspase-3 activity in GSCs (CoCl_2_ vs. control, *P* < 0.01; 14-3-3*β* siRNA vs. control, *P* < 0.001; CoCl_2_+14-3-3*β* siRNA vs. CoCl_2_, *P* < 0.01). (c) Effects of CoCl_2_ application and 14-3-3*β* siRNA transfection on Bcl-2 and Bax mRNA levels in GSCs. (d) Effects of CoCl_2_ application and 14-3-3*β* siRNA transfection on Bcl-2 and Bax expression in GSCs, Bax/Bcl-2 ratio was also calculated. (CoCl_2_ vs. control, *P* < 0.01; 14-3-3*β* siRNA vs. control, *P* < 0.001; CoCl_2_+14-3-3*β* siRNA vs. CoCl_2_, *P* < 0.01). *β*-Actin was used as a loading control.

The percentage of apoptotic cells was first evaluated by flow cytometry after 14-3-3*β* siRNA transfection or CoCl_2_ application. The results evidenced that 14-3-3*β* knockdown increased cell apoptosis; and the combination of CoCl_2_ application and 14-3-3*β* siRNA also induced a significant increase in cell apoptosis. Caspase-3 activity was investigated following CoCl_2_ application or 14-3-3*β* knockdown. The results demonstrated that caspase-3 activity was significantly decreased following CoCl_2_ application in GSCs; however, 14-3-3*β* knockdown increased caspase-3 activity (Figure 4). A rescue experiment also provided evidence that 14-3-3*β* siRNA application reversed the above effect of hypoxia induced by using CoCl_2_.

Furthermore, Bax and Bcl-2 mRNA levels were determined by qPCR. The results proved that CoCl_2_ treatment in GSCs decreased Bax mRNA level and increased Bcl-2 mRNA level, which was reversed when combined with 14-3-3*β* knockdown. In addition, after 14-3-3*β* knockdown, Bcl-2 mRNA level was reduced, whereas Bax mRNA level was increased (Figure 4). Western blotting results were used to observe Bax and Bcl-2 expression in GSCs, which are associated with the development of cell apoptosis. After CoCl_2_ application, the Bax protein expression in GSCs was reduced compared with that in the untreated group, whereas the Bcl-2 expression was increased. Furthermore, in the 14-3-3*β* siRNA transfection group, the Bax expression was increased, whereas the Bcl-2 expression was decreased. The Bax/Bcl-2 ratio, which expresses the apoptotic process of a cell, was therefore calculated. The results demonstrated that the decrease in Bax/Bcl-2 ratio after CoCl_2_ application was completely reversed following 14-3-3*β* knockdown.

### 3.5. Effects of PI_3_K Inhibition

**Figure 5:**
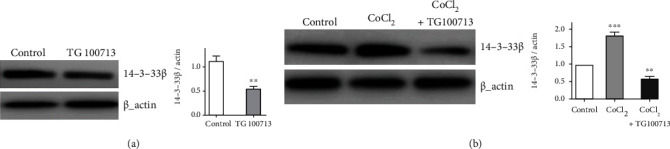
Effects of PI_3_K inhibition on the 14-3-3*β* expression. (a) Effects of *TG 100713* on the 14-3-3*β* expression, as assessed by western blotting. Densitometry analysis of 14-3-3*β*/actin (TG100713 vs. control, *P* < 0.01). (b) Effects of *TG 100713* and CoCl_2_ cotreatment on the 14-3-3*β* expression, as assessed by western blotting. Densitometry analysis of 14-3-3*β*/actin (CoCl_2_ vs. control, *P* < 0.001; CoCl_2_ + TG100713 vs. CoCl_2_, *P* < 0.01). *β*-Actin was used as a loading control.

In the following experiments, the pan isoform inhibitor of PI_3_K *TG 100713* was used to observe the association between hypoxia and 14-3-3*β*. The western blotting results confirmed that *TG 100713* application significantly reduced the 14-3-3*β* expression in GSCs. Furthermore, it was interesting that CoCl_2_ application did not enhance 14-3-3*β* expression in the presence of *TG 100713* (Figure 5). Hypoxia induced by using CoCl_2_ may promote therefore the 14-3-3*β* expression via the PI_3_K signaling pathway.

## 4. Discussion

Proteins of the 14-3-3 family are overexpressed in glioma, lung cancer, and astrocytoma and serve crucial roles in the biological behavior of various types of tumor [32, 33]. Furthermore, 14-3-3 regulates glioma cell proliferation through the glycogen synthase kinase 3/*β*-catenin signaling pathway [50, 51]. It has been reported that inhibition of HIF-1*α* by siRNA could reduce tumor growth, and hypoxic tumor microenvironment (TME) enhances tumor angiogenesis and progression [14, 15].

The results from the present study were as follows: (1) HIF-1*α* and 14-3-3*β* were highly expressed in GSCs and in tumor xenografts; (2) hypoxia induced by using CoCl_2_ enhanced 14-3-3*β* protein expression and mRNA levels in GSCs; (3) hypoxia promoted cell proliferation and associated PCNA expression, which was rescued by 14-3-3*β* knockdown; (4) tumor growth was significantly promoted by CoCl_2_ application, which was reversed following 14-3-3*β* knockdown; (5) cell apoptosis was increased after 14-3-3*β* siRNA application, which altered Bax/Bcl-2 ratio and caspase-3 activity in the hypoxic tumor microenvironment; and (6) hypoxia activated 14-3-3*β* via the PI_3_K signaling pathway. The present study therefore provided evidence that hypoxia may promote GSC growth via the PI_3_K signaling pathway both in vitro and in vivo by increasing 14-3-3*β* expression, which could be considered as a potential therapeutic target for patients undergoing clinical siRNA treatment.

Since glioma was characterized by high recurrence and low survival rate [3, 52], it is crucial to develop the novel strategies for the treatment of patients with glioma. The high HIF-1*α* expression was observed in the hypoxic tumor microenvironment (TME); thus, specific inhibition of HIF-1*α* may provide certain benefits in clinical cancer therapy when combined with a multitherapeutic approach. Previous studies also reported that 14-3-3*β* serves a key role in tumor angiogenesis and tumor formation, and that it could stimulate cell proliferation, cell cycle, and cell apoptosis to promotes tumor growth and progression [32–34]; however, few study has described the association between hypoxia and 14-3-3*β* modulation in glioma progression and metastasis and the biological behaviors of GSCs in vitro.

In the current experiments, the results demonstrated that both 14-3-3*β* and HIF-1*α* proteins were overexpressed in GSCs cells. Subsequently, the effect of hypoxia induced by using CoCl_2_, which can specifically enhance HIF-1*α* level [11, 12], on the 14-3-3*β* expression, was investigated in GSCs cells. The results provided the evidence that 14-3-3*β* protein expression and mRNA level were increased following hypoxia induced by using CoCl_2_ in a dose-dependent manner. Thus, it is hypothesized that 14-3-3*β*-induced GSC cell proliferation may be a potential downstream effect of hypoxia. Multitherapeutic approaches could therefore increase glioma therapeutic efficiency by combining 14-3-3*β* and HIF-1*α* inhibitors or siRNA application.

The results from cell proliferation experiments demonstrated that hypoxia promoted GSC cell proliferation by enhancing 14-3-3*β* expression level. These data indicated that HIF-1*α* and 14-3-3*β* may be associated in the tumor growth process. Additional evidence was obtained in the animal experiments by analyzing the survival rate and tumor volume. After 14-3-3*β* siRNA application, survival rate of the transplanted mice was enhanced, and tumor volume and weight were decreased, which indicated that the effect of hypoxia on tumor growth was reversed by 14-3-3*β* knockdown. In addition, a previous study also reported that 14-3-3*β* downregulation inhibits osteosarcoma cell proliferation and migration [53]. The present study therefore hypothesized that 14-3-3*β* may serve a crucial role in the hypoxia-induced tumor growth in glioma.

14-3-3*β* is highly expressed in various types of tumor and is associated with numerous human malignancies, including glioma, lung cancer, astrocytoma, breast cancer, squamous cell carcinoma, ovarian cancer, and liver cancer [32–35]. Tumor cell apoptosis is an important biological process associated with tumor growth. To clarify the underlying mechanism of hypoxia-induced tumor growth, the effect of CoCl_2_ application and 14-3-3*β* knockdown on GSC cell apoptosis was investigated. By observing the biomarker or apoptotic rate, the effect of hypoxia and 14-3-3*β* knockdown on cell apoptosis were evaluated both in vitro and in vivo. To do so, Bax/Bcl-2 ratio and caspase-3 activity were selected to reflect the changes in tumor apoptosis following the mentioned treatments by using 14-3-3*β* siRNA and CoCl_2_ application. Bax/Bcl-2 ratio was significantly decreased following CoCl_2_ treatment, which reduced tumor cell apoptosis. Furthermore, 14-3-3*β* knockdown enhanced GSC cell apoptosis via Bax/Bcl-2 ratio increase. The results from caspase-3 activity revealed similar tendencies. Above results suggested that hypoxia may enhance tumor growth or reduce tumor cell apoptosis via 14-3-3*β* modulation in GSCs. The underlying mechanism or downstream targets should be clarified in the further experiments.

The PI_3_K signaling pathway is closely associated with cell proliferation and cell apoptosis [23]. Hypoxia activates PI_3_K, which serves critical roles in cell survival and apoptosis [54, 55]. Furthermore, HIF-1*α* is known to relate to the Raf/MAPK/ERK axis, and hypoxia is a key event to leads to PI_3_K and Ras/MAPK signaling pathway activation [56]; however, whether hypoxia enhances 14-3-3*β* expression in GSCs via PI_3_K activation has not been investigated to date. In the present study, a specific inhibitor of PI_3_K reduced the 14-3-3*β* expression, even after CoCl_2_ application, which suggested that hypoxia may upregulate 14-3-3*β* via the PI_3_K signaling pathway. Hypoxia may therefore activate PI_3_K, which may promote 14-3-3*β* expression and influence the biological behavior of GSCs, including cell proliferation, survival, and apoptosis. PI_3_Ks, including the *α*, *β*, *γ*, or *δ* isoforms, are lipid kinases that serve crucial roles in the regulation of cell proliferation, cell cycle, and cell apoptosis [23]. The role of these different isoforms on cell proliferation, cell cycle, and cell apoptosis may be further investigated to clarify the underlying mechanism of the association between PI_3_Ks and 14-3-3*β*. Furthermore, it has been demonstrated that hypoxia induced HIF-1*α* modulates the communication between p53 and AKT-mTOR pathways in cancer cells [57–59]. The results from the present study also indicated that hypoxia may promote GSCs or glioma growth by activating the 14-3-3*β*. Subsequently, targeted inhibition of HIF-1*α* or 14-3-3*β* may provide novel clinical therapeutic strategies to treat patients with glioma.

14-3-3*β* and HIF-1*α* overexpression in tumors have been previously demonstrated [13, 32]. The present study therefore investigated whether 14-3-3*β* knockdown and PI_3_K inhibition could increase the therapeutic efficacy of glioma treatment in vitro and in vivo. In addition, since HIF-1*α* and the Raf/MAPK/ERK axis are associated, future works will investigate the changes between 14-3-3*β* and the Raf/MAPK signaling pathway. Through in vivo and in vitro experiments, the present study demonstrated that hypoxic tumor microenvironment (TME) may promote glioma progression via PI_3_K signaling pathway activation following the 14-3-3*β* overexpression. In conclusion, HIF-1*α* inhibition by using novel PI_3_K inhibitors combined with 14-3-3*β* knockdown by specific siRNA may represent an efficient therapeutic strategy to treat patients with glioma.

## Data Availability

The data used to support the findings of this study are included within the article.
